# Effect of Lifestyle or Metformin on Claims-Based Multimorbidity in the Diabetes Prevention Program Outcomes Study

**DOI:** 10.1093/geroni/igaf122.467

**Published:** 2025-12-31

**Authors:** Marcel Salive, Jesse Ames, Ashley Tjaden, Jill Crandall, Dana Dabelea, Helen Hazuda, Brandy Heckman-Stoddard, Elizabeth Venditti

**Affiliations:** National Institutes of Health, Rockville, Maryland, United States; The George Washington University Biostatistics Center, Rockville, Maryland, United States; The George Washington University Biostatistics Center, Rockville, Maryland, United States; Albert Einstein College of Medicine, Bronx, New York, United States; Lifecourse Epidemiology of Adiposity & Diabetes Center, Aurora, Colorado, United States; UT Health San Antonio, San Antonio, Texas, United States; National Cancer Institute Division of Cancer Prevention, Bethesda, Maryland, United States; University of Pittsburgh, Pittsburgh, Pennsylvania, United States

## Abstract

Studying how to prevent or delay not just one disease, e.g., diabetes, but multiple chronic conditions would have great potential for public health, yet few interventions have demonstrated success. We aimed to examine the impact of the lifestyle and metformin interventions, compared to placebo, on the long-term occurrence of multimorbidity. 3,234 adults at high risk of diabetes joined the DPP in 1996-1999 (mean age 51 years), were randomized to lifestyle, metformin, or placebo interventions, and followed through 2020. Consenting study participants’ data were matched with CMS data. Multimorbidity was defined as presence of ≥ 2 of 15 prevalent chronic conditions, as defined in CMS’ Chronic Condition Warehouse for fee-for-service claims and as adapted for Medicare Advantage encounters. Logistic regression models estimated the association between randomized treatment group and multimorbidity outcomes. Of 1,173 participants for whom Medicare data were available, 85% had ≥2 conditions (median=5). Odds of multimorbidity were lower among lifestyle participants compared to placebo (OR = 0.64; 95% CI = 0.42, 0.99) after adjustment for relevant covariates, but there were no differences between metformin and placebo participants. When restricting to high-cost dyad conditions, the results were similar (lifestyle vs. placebo: OR = 0.55; 95% CI = 0.35, 0.88). Increased odds of multimorbidity (excluding diabetes) were associated with the presence of diabetes, higher time-weighted HbA1c, and longer diabetes duration. Among a diabetes-related cohort, lifestyle intervention, not metformin, was associated with lower risk of multimorbidity >15 years after the end of original intervention. Multimorbidity was related to multiple diabetes measures.

